# Supplementary material for: Development of a prostate cancer biochemical recurrence risk signature using machine learning and motor protein-related genes
Source: PLoS One. 2025 Sep 16;20(9):e0332747. doi: 10.1371/journal.pone.0332747 (PMC12440175; doi:10.1371/journal.pone.0332747)
Supplement: Attachment — Submitted filename: Response to Reviewers.docx [file pone.0332747.s004.docx]

Dear editor and reviewers

Thank you for taking the time to review our manuscript titled "Development of a prostate cancer biochemical recurrence risk signature using machine learning and motor protein-related genes (PONE-D-25-09189)". We have carefully considered all of your feedback, and we are pleased to report that we have addressed each point raised in your review with the utmost attention to detail. Please note that the modifications in the revised draft are highlighted in red.

**Journal requirements:**

When submitting your revision, we need you to address these additional requirements.

1.  Please ensure that your manuscript meets PLOS ONE's style requirements, including those for file naming. The PLOS ONE style templates can be found at

[https://journals.plos.org/plosone/s/file?id=wjVg/PLOSOne_formatting_sample_main_body.pdf](https://track.editorialmanager.com/CL0/https://journals.plos.org/plosone/s/file?id=wjVg/PLOSOne_formatting_sample_main_body.pdf/1/010f0195cbd63b29-ece4f251-053a-48ce-b577-bd1e2dc8c723-000000/9xIpX94JdEnmwlGZl85Cl2ALsg8JQHf-EevydzCGZ5c=203)  and

[https://journals.plos.org/plosone/s/file?id=ba62/PLOSOne_formatting_sample_title_authors_affiliations.pdf](https://track.editorialmanager.com/CL0/https://journals.plos.org/plosone/s/file?id=ba62/PLOSOne_formatting_sample_title_authors_affiliations.pdf/1/010f0195cbd63b29-ece4f251-053a-48ce-b577-bd1e2dc8c723-000000/zg_kZCep27NVwWVta3_JH8YuqwU1ypliZWNesmQ8Tqs=203)

Response: We have revised the format of the title page and main text of the manuscript in accordance with the journal's requirements.

2. Please note that PLOS ONE has specific guidelines on code sharing for submissions in which author-generated code underpins the findings in the manuscript. In these cases, we expect all author-generated code to be made available without restrictions upon publication of the work. Please review our guidelines at https://journals.plos.org/plosone/s/materials-and-software-sharing#loc-sharing-code and ensure that your code is shared in a way that follows best practice and facilitates reproducibility and reuse.

RESPONSE: Our team is not willing to share the code of this study for the time being.

3. In the online submission form, you indicated that [The data that support the findings of this study are available from the corresponding author upon reasonable request].

All PLOS journals now require all data underlying the findings described in their manuscript to be freely available to other researchers, either 1. In a public repository, 2. Within the manuscript itself, or 3. Uploaded as supplementary information.

This policy applies to all data except where public deposition would breach compliance with the protocol approved by your research ethics board. If your data cannot be made publicly available for ethical or legal reasons (e.g., public availability would compromise patient privacy), please explain your reasons on resubmission and your exemption request will be escalated for approval.

Response: We have revised the Data Availability section at the end of the manuscript: The RNA expression profiles and corresponding clinical information used in this study are available in the TCGA (https://portal.gdc.cancer.gov), cBioPortal for Cancer Genomics (https://www.cbioportal.org/), and GEO (https://www.ncbi.nlm.nih.gov/geo/query/acc.cgi?acc=GSE70769) databases. All relevant data are within the manuscript and its Supporting Information files.

4. Please include your tables as part of your main manuscript and remove the individual files. Please note that supplementary tables (should remain/ be uploaded) as separate "supporting information" files

Response: We have inserted the Tables immediately after their first citation in the main text and uploaded the supplementary tables as separate "supporting information" files.

**Additional Editor Comments:**

1.The model performs well in TCGA-PRAD (AUC = 0.966) but poorly in MSKCC2010 (AUC = 0.739–0.684), suggesting potential dataset bias. Please address this issue.
Response: To address this issue, we first further added another cohort, GSE70769, which contains 92 prostate cancer patients and their follow-up information. We then used the sva package to remove batch effects among cohorts. After conducting machine learning analysis, we found that the problem mentioned by the reviewer still occurred based on the original optimal model selection criteria. Therefore, we adopted a new method: ranking the models according to the mean C-index in the validation cohort, and dividing the cohorts into high- and low-risk groups based on the median risk score. We selected models with a p-value less than 0.05 in the Log-Rank test between the high- and low-risk groups across all cohorts as the optimal models. Using this new selection method, we resolved the issue you raised and revised the subsequent results based on the new model.

2.The nomogram is developed using TCGA-PRAD but lacks external validation, which is essential to assess its reliability in broader populations.

Response: We further validated the reliability of the nomogram in the MSKCC2010 and GSE70769 cohorts. Using receiver operating characteristic (ROC) analysis, we calculated the AUC values for predicting 1-, 3-, and 5-year biochemical recurrence (BCR) in the above-mentioned cohorts.

3.While pathway enrichment analysis is performed, the study does not discuss how these pathways relate to prostate cancer progression or treatment response.

Response: We have thoroughly discussed the relationship between these pathways and prostate cancer progression or treatment response in the Discussion section of the revised manuscript.

**Reviewer comments**

**Reviewer #1:** Nice in silico exploratory study of motor protein transcriptomic signature and its correlation to biochemical recurrence in prostate cancer. Please if you could add additional paragraph to discussion section where you state certain limitations of the study, mainly that it is in silico and that as in any in silico study depending where you cut off the statistical significance in pathways enrichment analysis you get a different biological conclusions.

Response: We have addressed the limitations of the study in the final paragraph of the Discussion section as follows: While this in silico study provides novel insights into the prognostic value of MPRG expression signatures in prostate cancer, several important limitations must be acknowledged. First, the entirely computational nature of this investigation inherently limits biological interpretation, as we cannot confirm whether observed transcriptomic associations reflect functional biological mechanisms or correlative patterns. Second, pathway enrichment analysis remains inherently subjective due to arbitrary statistical thresholds for significance (e.g., FDR <0.05 vs FDR <0.1), which could lead to divergent biological interpretations depending on analytical parameters chosen by different researchers. Third, the retrospective design and reliance on TCGA data introduce inherent selection biases, necessitating validation in independent clinical cohorts with prospectively collected clinical outcomes. Fourth, while we identified candidate MPRGs associated with BCR, functional validation is required to establish causal relationships between these genes and prostate cancer progression. Finally, our nomogram model might benefit from inclusion of additional clinical variables such as PSA kinetics and radiographic findings to improve prognostic accuracy.

**Reviewer #2**: Thanks for the chance to review your work. This paper aimed to construct a risk signature of biochemical recurrence for prostate cancer, by using the methods of machine learning and the data from TCGA, KEGG, and MSKCC2010. Overall, this paper is solid and provided valuable insights for the understanding of Motor proteins and PCa progression. I have some minor suggestions for authors to refer to.
1) There are so many figures included in the paper, and please consider to conprehensively rearrange and reduce the number of all the figures. I think some figures should be merged into one.

Response: Based on the results of the re-analysis, we have combined Figures 2 and 3 to reduce the number of figures and have also revised some overlapping and unclear elements within the figures.
2) Please consider to add a table to summarize all the related studies.

Response: We have added a table (Table 2) in the Discussion section to summarize the studies on the related markers. Since there are many studies on the functions and mechanisms of these genes in cancer, we mainly summarized the functions and mechanisms of the eight MPRG marker genes in prostate cancer.

3) Some sentences are confusing to follow, and authors should carefully check all the typos and grammar.

Response: We have systematically reviewed the sentences throughout the manuscript to make them more comprehensible and have also corrected some spelling and grammatical errors. Specifically:

Changed "being" to "and is" on line 47.

Changed "role" to "importances" on line 19.

Added "thus" on line 58.

Added two commas on line 62.

Changed "could" to "may" on line 75.

Changed "along with" to "as well as" on line 286.

Changed "include" to "incorporates" on line 303.

Added "and" on line 360.

Added "and/" on line 368.

Added "for improving outcomes" on lines 369-370.

In addition, we have checked and proofread the sections modified due to other comments for grammatical and phrasing issues.
