# Supplementary material for: Development of a prostate cancer biochemical recurrence risk signature using machine learning and motor protein-related genes
Source: PLoS One. 2025 Sep 16;20(9):e0332747. doi: 10.1371/journal.pone.0332747 (PMC12440175; doi:10.1371/journal.pone.0332747)
Supplement: Attachment — Submitted filename: Response to Reviewers - 2.docx [file pone.0332747.s005.docx]

Dear editor and reviewers

Thank you for taking the time to review our manuscript titled "Development of a prostate cancer biochemical recurrence risk signature using machine learning and motor protein-related genes (Manuscript ID: PONE-D-25-09189R1)". We have carefully considered all of your feedback, and we are pleased to report that we have addressed each point raised in your review with the utmost attention to detail. Please note that the modifications in the revised draft are highlighted in red.

Journal Requirements:

Please review your reference list to ensure that it is complete and correct. If you have cited papers that have been retracted, please include the rationale for doing so in the manuscript text, or remove these references and replace them with relevant current references. Any changes to the reference list should be mentioned in the rebuttal letter that accompanies your revised manuscript. If you need to cite a retracted article, indicate the article’s retracted status in the References list and also include a citation and full reference for the retraction notice.

Response: We have reviewed the references to ensure they meet the requirements.

Additional Editor Comments:

Please address my requests below in addition to the reviewer comments:

1) While external cohorts were included, the conclusions rely entirely on bioinformatics analyses. Experimental or clinical validation would substantially strengthen the findings. Please update your manuscript to include further validation of your findings, and ensure that cohort matching on clinical covariates is fully addressed.

Response: In this revision, we have supplemented the in vitro gene expression analysis, comparing the expression of MPRGs between PCa cell lines DU145 and LNCaP and the benign prostate hyperplasia epithelial cell line BPH-1, further validating the results of the bioinformatics analysis.

2) Although key clinical variables such as T and N stages were analyzed, important factors like PSA levels, treatment history, and comorbidities were not included or discussed. Please incorporate these to improve the robustness of the prognostic model.

Response: In this revision, we have included PSA and treatment history in the analysis. The results show that PSA is also an independent prognostic factor for prostate cancer. We have refined the nomogram and its performance evaluation results. Since the MSKCC cohort does not have PSA data, we have only validated the performance of the nomogram in the GEO cohort. Please refer to Table 1, Fig 7, and the section on Construction of Nomogram Using MPRG-Derived Risk Score for detailed results.

Reviewer #6: The Manuscript presented by Weixing Wang et al entitled: "Development of a Prostate Cancer Biochemical Recurrence Risk Signature Using Machine Learning and Motor Protein-Related Genes" is technically sound and presents a comprehensive bioinformatics and machine learning pipeline to develop a prognostic model for biochemical recurrence (BCR) in prostate cancer based on motor protein-related genes (MPRGs).

1. Technical soundness:
The use of two independent cohorts (TCGA-PRAD and MSKCC2010) for training and validation strengthens the reliability of the findings. the study's conclusions are generally well-supported by the presented data, including differential gene expression, survival analysis, ROC curves, immune cell infiltration correlations, and drug sensitivity predictions. However, the biological interpretation of some genes within the 8-MPRG signature could benefit from more mechanistic explanation or references, especially for less characterized genes in prostate cancer context.
Response: Thank you very much for your comments. However, you should be reviewing the initial version, as some of the key issues you mentioned have already been revised in the first revision. Regarding this issue, in the first revision, we have conducted an exhaustive literature analysis and organization of the 8 MPRGs, which have been compiled in Table 2.

2. statistical analysis:
The statistical approach is appropriately rigorous, especially with the incorporation of 10 machine learning algorithms and 101 model combinations. The authors applied uninvariate Cox regression, leave-one-out cross-validation (LOOCV), and evaluated performance using Harrel's index and ROC/AUC analysis- all of which are acceptable and correctly used.
That said, some aspects could be strengthened:
- the model calibration could be described more clearly. Were the calibration curves statistically evaluated?
- Multivariate Cox regression was performed, but the interpretation that riskscore is the only independent predictor (Table 1) requires further context (e.g., confidence intervals, hazard ratios).
Response: In our analysis, we used the rms::calibrate() function for calibration curve analysis, which is adopted by the majority of similar studies. This function does provide statistical evaluation in a Cox model (fitted by cph()), but these evaluations are indirect and quantitative, rather than a single p-value or χ² statistic like the Hosmer-Lemeshow (HL) test applicable to generalized linear models. It mainly assesses the model's calibration performance by generating a calibration object, focusing on correcting overfitting and quantifying prediction bias. It uses bootstrapping or cross-validation methods to estimate the model's optimism, generates calibration curves, and combines visual inspection for analysis. We have added these details to the Materials and Methods section. Additionally, we have further described the details of the calibration curve in the Results section. Moreover, we have re-analyzed the data according to the reviewers' and editors' comments, and the new results incorporating the risk score, T stage, and PSA data have been used to construct the nomogram. Therefore, the updated Cox regression results have also been revised.
3. Manuscript presentation and language:
The manuscript is generally intelligible and logically structured, with standard scientific English. However, there are numerous grammatical and typographical issues that should be addresssed to improve clarity. Some sentences are overly long and awkwardly constructed.
Examples:
- Line 13: "but their involvement in the biochemical recurrence (BCR) of prostate cancer is not well understood" can be improved as " but their role in biochemical recurrence (BCR) of prostate cancer remains unclear."
- Line 183: Age was observed to be positively associated with the riskscores (r=0.16, p=0,0053, Figure 4D), indicating that older patients had significantly higher risk scores compared to younger patients (Figure 4E)." can be improved as " Age was positively correlated with risk scores (r=0.16, p=0.0053; Figure 4D), suggesting that older patients tend to have higher riskscores than younger individuals (Figure 4E)."
- Line 23: Consider revising "with 8 significantly associated with BCR" to "of which 8 were significantly associated with BCR."
Response: In this revision, we have made the changes as per your suggestions, systematically checked and corrected spelling errors and grammatical mistakes in the manuscript, and improved some sentences that were too long and awkwardly constructed.

- Figure Legends are repetitive; consider condensing for example:
Figure 3 legend: this legend restates what each panel obviously shows, without adding interpretive value or clarification. it's descriptive but nor insightful. it can be improved as " Performance of the MPRG-based RSF model in predicting BCR. Risk score distributions, Kaplan-Meier survival curves, and ROC analyses for 1-, 3-, and 5-year BCR prediction are shown for the TCGA-PRAD (A-C) and MSKCC2010 (D-F) cohorts. Please consider same condensing to other legends as well.
Response: We have streamlined the figure legends as per your suggestion.

- Data availability:
Refer to (Line328-330): "The data that support the findings of this study are available from the corresponding author upon request." However, earlier in the form-based metadata (near the beginning of the PDF), the authors checked "Yes- all data are fully available without restriction." this is inconsistent and contradicts PLOS ONE's open data policy. As the data appear to be sourced from public databases (e.g., TCGA, cBioPortal), the authors should revise statement to include direct links or accession numbers to these sources and clearly indicate that no restrictions apply.
Response: We have revised the data availability paragraph and provided the link for data access.

- Novelty and impact:
The manuscript offers techical novelty through its use of motor protein-related genes in a machine learning framework to predict biochemical recurrence in prostate cancer. while the computational framework id rigorous and the biological rationale is sound, the novelty would be strengthened by comparing this model against existing BCR signatures and discussing its added clinical value over current available tools. Moreover, the lack of experimental validation or prospective cohort analyses limits the immediate applicability of the model in clinical settings.
Response: In this revision, we have conducted in vitro analysis of the gene expression of the 8 MPRGs to further validate the results of the bioinformatics analysis.
